# Perceived Motivational Climates and Employee Energy: The Mediating Role of Basic Psychological Needs

**DOI:** 10.3389/fpsyg.2020.01509

**Published:** 2020-07-10

**Authors:** Christina G. L. Nerstad, Marjolein C. J. Caniëls, Glyn C. Roberts, Astrid M. Richardsen

**Affiliations:** ^1^Oslo Business School, OsloMet – Oslo Metropolitan University, Oslo, Norway; ^2^Department of Leadership and Organizational Behavior, BI Norwegian Business School, Oslo, Norway; ^3^Faculty of Management, Open Universiteit, Heerlen, Netherlands; ^4^Institute of Sport and Social Sciences, Norwegian School of Sport Sciences, Oslo, Norway

**Keywords:** mastery climate, performance climate, autonomy, competence, relatedness, emotional exhaustion, vigor

## Abstract

This study draws on achievement goal theory and self-determination theory to examine the associations among two motivational climates (i.e., mastery and performance) and two indicators of energy at work (i.e., vigor and emotional exhaustion), as well as the mediating role of basic psychological need satisfaction (i.e., autonomy, relatedness, and competence). A two-wave longitudinal study was conducted collecting data from 1,081 engineers and technologists. We applied previously validated instruments to assess the variables of interest. Structural equation modeling analyses were conducted to test the hypotheses. Our findings show that mastery climate is positively and significantly related to each of the three basic needs, whereas a performance climate is negatively and significantly related to each of the three basic needs. Further, the results indicate that the basic needs are significantly associated with both measures of energy, negatively with emotional exhaustion and positively with vigor. This pattern of results suggests that basic psychological need satisfaction mediates the relationship between motivational climates and energy at work.

## Introduction

Technological innovations and advances have facilitated the possibility that employees may be “at work” at virtually any time. The increasing organizational emphasis on longer working hours, striving toward excellence, striving for perfection, and competition with coworkers, often resulting from globalization and technology advances, can affect the energy of employees (i.e., vigor and emotional exhaustion) at work (Nerstad et al., 2013b; Di Fabio, 2017). A sustainable organization has the capacity to be productive and endure over time and, therefore, in addition to economic and environmental aspects, requires a consideration of a human dimension of sustainability—employee energy at work (Brown, 1999; Fritz et al., 2011). In this article, we explore environmental determinants of employees’ energy—vigor and emotional exhaustion—as well as important mechanisms contributing to such processes at work.

Employees may differ in the amount of energy they display, and their energy can vary on a continuum, from vigor to emotional exhaustion (Schaufeli and Salanova, 2007). Vigor is an important dimension of work engagement—defined as a work-related positive and fulfilling state of mind—and is characterized by having high levels of mental resilience and energy while working, persistence even in the face of difficulties, and a willingness to invest effort in one’s work (Schaufeli and Bakker, 2004a; Schaufeli and Salanova, 2007).

Emotional exhaustion, on the other hand, is a major component of burnout and is a multidimensional psychological syndrome reflecting individual responses to interpersonal and emotional stressors at work (Maslach and Jackson, 1981; Swider and Zimmerman, 2010). Emotional exhaustion is characterized as a state of being drained of emotional energy and the experience of feeling that one has inadequate emotional resources to handle the situation (Maslach, 1982). Such energy depletion may be described as a human energy crisis (Fritz et al., 2011). Vigor and emotional exhaustion are seen as opposite indicators of the energy dimension of occupational well-being (Maslach and Leiter, 1997; Schaufeli and Salanova, 2007).

Empirical evidence has shown that these energy dimensions—vigor and emotional exhaustion—show a weak to moderate correlation (Gonzaléz-Roma et al., 2005; Halbesleben, 2010). Moreover, meta-analytic and other empirical findings suggest that vigor tends to be related to adaptive outcomes, such as increased performance, important resources at work (e.g., feedback), physical health, and subjective work capacity (Shirom et al., 2008; Halbesleben, 2010; Shirom, 2010). On the other hand, the converse emotional exhaustion tends to predict maladaptive outcomes, such as reduced physical health, subjective work capacity, job satisfaction, organizational commitment, and higher turnover intentions (Lee and Ashforth, 1996; Shirom et al., 2008; Lee et al., 2011). Given that vigor represents positive functioning and well-being at work, whereas emotional exhaustion represents the opposite (ill-being), it is important to further clarify and understand the antecedents and possible pathways leading to vigor and/or emotional exhaustion (Shirom, 2010; Aronsson et al., 2017).

In this study, we therefore draw upon two prominent motivational theories—achievement goal theory (AGT; Nicholls, 1989; Ames, 1992b) and self-determination theory (SDT; Deci and Ryan, 2000; Gagné and Deci, 2005)—to argue that the perceived motivational climate at work, as defined by AGT, affects employee need satisfaction, as defined by SDT, and may in turn play an important role in predicting employees’ energy at work.

The characteristics of the environment and the situations that influence the way in which individuals perceive the extant achievement criteria of success and failure forms what Ames (1992a) calls the motivational climate (mastery climate and performance climate). Empirical findings have shown that a mastery climate typically predicts positive outcomes, such as enjoyment, engagement, autonomous motivation, and knowledge sharing, whereas a performance climate predicts less beneficial outcomes, such as controlled motivation, burnout, negative affect, and performance anxiety (e.g., Ntoumanis and Biddle, 1999; Abrahamsen et al., 2008; Lemyre et al., 2008; Nerstad et al., 2013a, 2018; Harwood et al., 2015; Buch et al., 2017). Still, the current literature on employee energy has not yet clarified the mechanisms by which a perceived motivational climate leads to energy. Given that a mastery climate values aspects such as autonomy, positive interdependence among employees, and skill development, it is likely to predict an employee’s basic psychological need satisfaction—the fulfillment of needs for autonomy, belongingness, and competence (cf. Ntoumanis and Biddle, 1999; Quested and Duda, 2009). A performance climate values control, normative competence, and intrateam competition and is therefore likely to reduce an employee’s need satisfaction (Ntoumanis and Biddle, 1999). Individuals are more likely to experience ill-being when they report low levels of need satisfaction, but higher levels of well-being when they report higher levels of need satisfaction (Quested and Duda, 2011, 2009; Vansteenkiste et al., 2020). We therefore propose that a perceived mastery climate is likely to facilitate a health-enhancing process, by increasing employees’ vigor through their satisfaction of needs. By contrast, a perceived performance climate is likely to facilitate a health-impairing process, by increasing emotional exhaustion through a reduction of need satisfaction.

Our theoretical point of view and empirical findings represent a significant contribution to the overall occupational health psychology literature, which includes burnout and work engagement literatures. In general, occupational health psychology has been challenged by the question of how to construct “healthy” organizations and thereby create working environments that enhance employee work-related health and well-being over time (Cooper et al., 2001; Di Fabio, 2017). As many of today’s organizations experience a high occurrence of depleted energy (e.g., emotional exhaustion) among their employees and a need for more knowledge on the psychosocial work environments that can prevent burnout dimensions and enhance well-being, more research on the antecedents of emotional exhaustion and vigor is needed (Swider and Zimmerman, 2010; Fritz et al., 2011; Shirom, 2011; Ten Brummelhuis et al., 2011; Aronsson et al., 2017). Further, scholars have called for research that fits the dimensions of work engagement (e.g., vigor) with other theories of motivation than those that have already been clarified (Meyer and Gagné, 2008; Christian et al., 2011). Our study intends to contribute to the literature by answering such calls to clarify environmental determinants and mechanisms of future energy from a motivational point of view (Meyer and Gagné, 2008; Shirom, 2010; Aronsson et al., 2017; Vansteenkiste et al., 2020). Thus, it is expected that a perceived motivational climate impacts future energy through employee need satisfaction.

In practice, our research should provide guidance to organizations and their leaders regarding how they can enable employees to sustain their energy and thus facilitate their well-being at work.

## Theory and Hypotheses

### Employee Energy and the Perceived Motivational Climate

Emotional exhaustion can be understood as the core component of burnout and indicates feelings of strain, particularly chronic fatigue, frustration, and loss of energy (Demerouti et al., 2001; Schaufeli et al., 2009; Seidler et al., 2014). Given that employees feel that their resources are insufficient to deal with the work situation, the emotional exhaustion dimension of burnout “clearly places the individual strain experience within the social context of the workplace” (Maslach and Leiter, 2008, 498). Employee depletion of energy resources typically arises from prolonged exposure to stressors that exceed a person’s resources to cope or when valued resources are lost (Lee and Ashforth, 1996; Cooper et al., 2001). Meta-analytical evidence has shown that high demands, job insecurity, low job control, low reward, and high work load seem to increase the risk of exhaustion (e.g., Seidler et al., 2014; Aronsson et al., 2017).

Emotional exhaustion has been theorized to be the opposite pole of vigor—defined as employee feelings of having high levels of energy, persistence, cognitive liveliness, and resilience—on what may be characterized as the *energy continuum* (Schaufeli and Salanova, 2007; Shirom, 2010). Such a continuum indicates that feelings of fatigue and exhaustion may not be experienced at the same time as mental energy and resiliency (Mäkikangas et al., 2017). This means that emotional exhaustion represents employee strain and ill health, whereas vigor represents employee well-being (Schaufeli and Salanova, 2007; Shirom, 2010; Mäkikangas et al., 2017). Extant research suggests that important predictors of vigor include job characteristics (e.g., job and task significance, task identity, feedback from supervisors, and achieving success), high-quality connections with others (empathic listening, learning from one another), leadership style (leaders’ positive affect, relationship building), group resources (mutual trust, social support), and organizational resources (rest and recuperation during work) (Shirom, 2011).

The work engagement and burnout literature emphasizes the relevance of context as an important determinant of employees’ energy—emotional exhaustion and vigor (Schaufeli and Salanova, 2007; Maslach and Leiter, 2008). Although the vast amount of research (e.g., Van den Broeck et al., 2008; Shirom, 2010) has focused on job demands and resources as vital antecedents of employee energy, the motivational (psychological) climate at work, as defined by AGT (Nicholls, 1989; Ames, 1992b), represents another relevant contextual determinant (cf. Lemyre et al., 2008; Nerstad et al., 2013a). According to AGT, the perceived motivational climate may be identified as personal perceptions of the extant criteria of success and failure, emphasized through policies, practices, and procedures in the work environment (Nerstad et al., 2013a). It is represented by two types of climate, a mastery climate or a performance climate. A mastery climate exists when criteria of success, characterized by aspects such as self-learning, cooperation, task-mastery, development, and effort, are supported in the work situation (Nicholls, 1989; Ames, 1992b; Nerstad et al., 2013a). A mastery climate is suggested to promote more adaptive behavior, such as trying hard and persisting when faced with difficulties, and to promote well-being (Ntoumanis and Biddle, 1999; Harwood et al., 2015; Roberts et al., 2018). A performance climate, on the other hand, exists when criteria of success are characterized by the importance of demonstrating ability through normative comparisons. A performance climate is suggested to promote more maladaptive behavior, such as seeking easy tasks and giving up when faced with difficulties, and may promote ill health (Ames, 1992b; Roberts and Nerstad, 2020).

The individual’s perceptions of these climates, based on prior experiences, determine which of the climates is seen as the most appropriate in a specific context. Because of this, but also because individuals are seen as active participants in their own socialization (Hewstone and Stroebe, 2001), the same motivational climate may be perceived quite differently by individual employees. According to AGT, individuals who perceive a mastery climate are likely to experience adaptive outcomes, whereas maladaptive outcomes are expected for those individuals who perceive a performance climate (Roberts and Nerstad, 2020).

Studies have demonstrated that the motivational climate is directly associated with perceptions of well-being (e.g., Reinboth and Duda, 2004; Lemyre et al., 2008; Quested and Duda, 2009; Nerstad et al., 2013a). For example, Reinboth and Duda (2004) found that athletes’ perceptions of a performance climate were positively related to burnout, but that perceptions of a mastery climate were not. These findings were also supported by another study, which indicated that individuals’ perceptions of a mastery climate may temper perceptions of burnout (Smith et al., 2010). According to AGT (Nicholls, 1989; Ames, 1992b), mastery and performance climates influence cognitive, affective, and behavioral outcomes in different ways, because they represent different conceptions of success. It is therefore likely that mastery and performance climate perceptions at work have a different direct influence on vigor and emotional exhaustion over time. Based on the relevant theory and empirical findings, we expected the present study to show that a perceived mastery climate has a direct negative relationship with future emotional exhaustion, but a direct positive relationship with future vigor (7-month time lag). By contrast, we expected a performance climate to have a direct negative future relationship with vigor, but a direct positive future relationship with emotional exhaustion (7-month time lag).

### The Mediating Role of Basic Psychological Need Satisfaction

Self-determination theory (Deci and Ryan, 2000) is a general theory of human motivation that applies to several domains, including work, sport, education, and health. SDT posits that, when three basic psychological needs—the need for competence, the need for autonomy, and the need for relatedness—are satisfied, individuals will be well motivated and experience well-being (Deci and Ryan, 2000; Van den Broeck et al., 2016; Vansteenkiste et al., 2020). When employees’ needs are satisfied, they are more likely to be more autonomously motivated and experience improved well-being, psychological energy, and health (Ng et al., 2012; Li et al., 2019). Thus, basic psychological needs are the most important constructs within SDT (Deci and Ryan, 2000; Van den Broeck et al., 2016).

The need for competence has been characterized as an individual’s striving to exercise and express abilities; thus, one has a need to experience a sense of mastery and confidence in action (Deci and Ryan, 2002; Vansteenkiste et al., 2020). Such a need is satisfied when individuals experience the opportunity to extend their expertise and skills (Vansteenkiste et al., 2020). The need for autonomy has been defined as an individual’s efforts to determine his/her own behavior and act based on interest and integrated values (Deci and Ryan, 1985). Individuals who have this need satisfied experience a sense of integrity because their emotions, cognitions, and behaviors are authentic and self-initiated (Vansteenkiste et al., 2020). Last, the need for relatedness characterizes individuals’ attempts to have a coherent and satisfying involvement with others (Deci and Ryan, 2002). Such a need is satisfied when individuals experience a sense of communion, develop close relations, and feel connected to others (Van den Broeck et al., 2016). In line with SDT’s assumptions, a great amount of empirical research indicates support for the positive association between satisfaction of the three needs and attitudes, behavior, and well-being (Deci and Ryan, 2000; Gagné and Deci, 2005; Van den Broeck et al., 2016; Deci et al., 2017).

**FIGURE 1:**
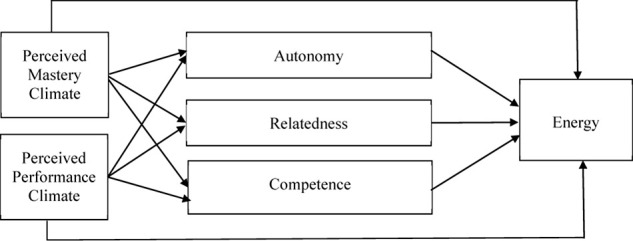
Conceptual model.

Self-determination theory posits that social–environmental factors influence employees’ motivation and experiences through the mediating variables of basic psychological needs for autonomy, competence, and relatedness (Vallerand, 1997; Gagné and Deci, 2005; Deci et al., 2017). Further, social conditions that give opportunities to satisfy the three fundamental psychological needs are predicted to facilitate energy and well-being (Ng et al., 2012; Vansteenkiste et al., 2020). Mastery and performance climates represent such social conditions that may contribute to either enhance or reduce need satisfaction (Ntoumanis and Biddle, 1999) and in turn influence employees’ energy (see Figure 1). A mastery climate facilitates the autonomy of behavior, evaluates success in a self-referential manner, and reinforces personal progress and the sense that each employee has an important role within the work group (Quested and Duda, 2009). When employees experience a mastery climate, their motivation is derived from the intrinsic properties of their work tasks and not from the expected outcomes (e.g., attainment of rewards, public recognition, social approval, demonstration of normative ability) (Ntoumanis, 2001; Buch et al., 2017). Rather, individuals experience satisfaction while they strive to grow, master, and learn. Their needs for competence, autonomy, and relatedness are likely to be satisfied, because such a climate values volition, self-referent development of competence to achieve desired outcomes, and meaningful interpersonal relationships (Reinboth and Duda, 2004; Quested and Duda, 2009). In turn, because greater opportunities for the satisfaction of the three needs are provided, employees are likely to experience well-being and reduced ill-being (Ng et al., 2012; Vansteenkiste et al., 2020). For example, one study in a sports setting found that a mastery climate predicted increased subjective vitality through the satisfaction of the three basic psychological needs (Reinboth and Duda, 2006). Also, Gagné et al. (2003) found that daily need satisfaction predicted increased daily well-being (i.e., subjective vitality and self-esteem) among young female gymnasts. When employees perceive a mastery climate and their needs in turn are satisfied, their emotional exhaustion is likely to be reduced—given their feelings of well-being (Quested and Duda, 2011). We therefore hypothesize the following:

**Hypothesis 1:** Basic needs satisfaction mediates the positive relationship between a perceived mastery climate and vigor.

**Hypothesis 2:** Basic needs satisfaction mediates the negative relationship between a perceived mastery climate and emotional exhaustion.

By contrast, if the work context is characterized by controlling features—forced interpersonal rivalry among employees, normative ability comparison, pressure to perform better than colleagues, judgment of progress based on normative standards, and so on—of a performance climate, the satisfaction of the needs for competence, autonomy, and relatedness is likely to be undermined (Reinboth and Duda, 2006; Quested and Duda, 2009). Specifically, a performance climate is likely to lead employees to become more interested in the anticipated outcomes of their work rather than the work itself and developing the ability to achieve desired outcomes (Ntoumanis, 2001). Attaining rewards and social approval, demonstrating superior competence, and striving for public recognition may control their behavior and prevent them from developing meaningful interpersonal relationships (Ntoumanis, 2001; Quested and Duda, 2009). As a result, employees are less likely to find personal satisfaction in the inherent aspects of their work, which may result in a depletion of their energy (reduced vigor and increased emotional exhaustion).

To our knowledge, no other study has investigated the mediating role of need satisfaction in the performance climate–emotional exhaustion relationship. However, one study (Quested and Duda, 2011) tested the relationship between need satisfaction and athlete emotional and physical exhaustion, without finding support for such a relationship. This is surprising and contrary to what is expected, based on theory (AGT and SDT), namely, that a performance climate reduces need satisfaction, which in turn depletes employees’ psychological energy—reduces vigor/enhances emotional exhaustion (Ntoumanis, 2001; Quested and Duda, 2011; Ng et al., 2012). Drawing on motivational theory, it is therefore important to further clarify the mediating role of psychological needs in such a relationship. We therefore hypothesize the following:

**Hypothesis 3:** Basic needs satisfaction mediates the negative relationship between a perceived performance climate and vigor.

**Hypothesis 4:** Basic needs satisfaction mediates the positive relationship between a perceived performance climate and emotional exhaustion.

## Materials and Methods

### Sample and Procedure

To ensure that ethical standards were met, information about the study’s design, planned sample and procedure, and the questionnaires were evaluated and approved by the Norwegian Social Science Data Services. Approximately 33,275^1^ Norwegian engineers and technologists representing different occupational divisions [research and development, information technology (IT), health, safety and the environment, human resource management, consultancy, laboratory, logistics, production, building and reconstruction, sales and marketing, services and economy] and various organizations situated all over Norway were invited to participate. The study was conducted in collaboration with the participants’ union, and the union was responsible for distributing the questionnaire to members through a web-based tool (QuestBack). The study was longitudinal, with measurements at two time points. The time interval was 7 months. At T1, we received 8,282 completed responses, achieving a response rate of approximately 25%. The Time 2 (T2) data collection resulted in 4,040 completed responses, representing a response rate of approximately 49%. Because of a technical problem with the web-based tool, it was possible to match only 1,081 of the respondents. Consequently, we conducted an independent-sample *t*-test to determine whether there were any differences between the 2,959 respondents that we were unable to match and the 1,081 remaining respondents. The *t*-test results indicated that there were some significant demographical differences in gender, education, and hours worked per week; however, there were no significant differences regarding the other study variables. We therefore controlled for the listed demographic variables in all analyses (*N* = 1,081).

To ensure that the respondents were representative of the total sample, we also compared their demographic variables to the union’s statistics regarding member-specific demographic variables. These statistics are constantly updated by the union. When comparing the demographic variables of this study sample with the overall statistics of the union (i.e., age, gender, work sector), the participants seemed to be representative of the total union population (i.e., approximately 66,000 members in total; age: mean = 46.8 years; gender: 78% male; work sector: 58% private sector; 32% public sector). Of the total sample (T1), 75% were men, 53% worked within the private sector, 32% were public sector employees, 83% were married or had a life partner, and 85% had a university degree. Further, the mean number of years that an employee had been in his/her present position was 3.35 (SD = 0.89), and the mean number of weekly working hours was 40.45 (SD = 6.06).

The composition of gender in the union overall but also in our sample seems very unbalanced. This lack of balance may possibly be linked to the various areas of work represented in the sample (and in the union). Table 1 shows the distribution of gender based on the respective work area. As indicated in Table 1, the work areas particularly dominated by men are “project work/building and construction services,” “planning and logistics,” and “IT/data services.” The only work area that is dominated by females in this sample is laboratory work. It should be noted that the list of work areas was developed by the union to be relevant for study participants.

**TABLE 1:** Gender distribution according to work area.

Work area	Males	Females	Total
Research/development	317	148	465
HMS/quality security	242	139	381
IT/computer facilities	987	213	1,200
Consultant/advisory	593	165	758
Laboratory work	114	609	723
Training/personnel work	142	50	192
Planning/logistics	246	69	315
Production/operations	1,185	146	1,331
Project work/building and construction services	1,461	264	1,725
Sales/marketing	243	44	287
Economy	24	9	33
Other/don’t know	632	240	872
Total	6,186	2,096	8,282

### Measures

Multiple-item scales, closely following previous studies, were used to measure each construct. All variables were measured at two moments in time (T1 and T2) with a 7-month lag. The survey covered the following construct variables.

#### Emotional Exhaustion

Emotional exhaustion, as the leading symptom of burnout, was measured by five items from the Norwegian version (Richardsen and Martinussen, 2004) of the Maslach Burnout Inventory–General Survey developed by Maslach and Jackson (1981) and Maslach et al. (1996). We used a seven-point Likert-type scale, ranging from “never in the past year” (0) to “every day” (7). A sample item included “I feel emotionally drained from my work” (α = 0.88).

#### Vigor

Vigor was assessed with three items from the vigor dimension of the nine-item Dutch Utrecht Work Engagement Scale (UWES, or UBES in Dutch; Schaufeli and Bakker, 2004b) (α = 0.91). An example item is “At my work, I feel bursting with energy.” Items were rated on a seven-point scale, ranging from 1 (never) to 7 (daily). Validity and reliability of this scale have been confirmed extensively in previous research (Schaufeli et al., 2006; Seppälä et al., 2009).

#### Perceived Motivational Climate

Perceived motivational climate was measured using 14 items developed and validated by Nerstad et al. (2013a). The scale asks respondents how employees perceive success to be defined in their work situations. The extent to which a performance climate is present is assessed with eight items (e.g., “In my department/work group, it is important to achieve more than others”) (α = 0.83), and the extent to which a mastery climate is present is assessed with six items (e.g., “In my department/work group, one is encouraged to cooperate and exchange thoughts and ideas mutually”) (α = 0.86). The items were scored on a five-point Likert scale, ranging from 1 (strongly disagree) to 5 (strongly agree).

#### Basic Psychological Need Satisfaction

*Basic psychological need satisfaction at work* was measured with a scale originally developed by Kasser et al. (1992) and further developed by Ilardi et al. (1993) and Deci et al. (2001). The scale consists of 21 items that represent three subdimensions: (1) autonomy (seven items; α = 0.68), for example, “I feel like I can make a lot of inputs to deciding how my job gets done”; (2) relatedness (eight items; α = 0.82), “I really like the people I work with”; and (3) competence (six items; α = 0.70), for example, “People at work tell me I am good at what I do.” Items were measured on a seven-point Likert scale ranging from 1 (not true at all) to 7 (very true).

#### Controls

We assessed several control variables, as prior research suggests that the demographic background of employees may account for the variance in their energy level (e.g., Bakker et al., 2005). Age was measured in years. Tenure was measured by years of experience in the current or similar function. The respondents were asked to report their gender based on a dichotomous variable, in which 1 represented men, and 2 represented women. They were also asked to report their level of education on a five-item scale, where 1 represented high school, 2 represented vocational school, 3 represented college, 4 represented a university degree, and 5 represented the category “other.”

### Analytical Strategy

We conducted a confirmatory factor analysis (CFA) using the R packages semTable (Johnson and Kite, 2019) and semTools (Jorgensen et al., 2018; Peters, 2019), which are based on Lavaan (Rosseel, 2012) to determine item retention and to secure discriminant validity (Farrell, 2010). Our research model consisted of seven latent variables—perceived mastery climate, perceived performance climate, the need for autonomy, the need for competence, the need for relatedness, emotional exhaustion, and vigor. To be certain, we compared the fit statistics of such a correlated-traits model with seven factors with the fit statistics of one, three, four, five, and six factors. To assess model fit of each of the competing models, we explored various fit indices, recommended by Kline (2005) and Byrne (2013). An acceptable fit is indicated by a standardized root mean square residual (SRMR) value of less than 0.80 and a root mean square error of approximation (RMSEA) value of less than 0.08 (Kline, 2005; Byrne, 2013). According to Hu and Bentler (1999), a comparative fit index (CFI) value and Tucker–Lewis index (TLI) value of 0.90 or greater can be considered to indicate an “acceptable” model fit. We also report the goodness-of-fit index (GFI) for which a cutoff point of 0.90 has been recommended (Marsh, 2007).

To examine the hypothesized mediation influence, we conducted structural equation modeling using the R package Rosetta (Peters, 2019), which is based on Lavaan (Rosseel, 2012) and facilitates bootstrapping. We checked for robustness of the model by investigating alternative specifications, such as using mediator variables at T2 and controlling for the dependent variables at T1. These analyses indicated that our findings are robust to alternative model specifications.

## Results

### Descriptive Statistics

Table 2 shows the means, standard deviations, and correlations between the main variables in our study. We observe that the control variables do not structurally associate with any of the main variables, as all correlations are less than 0.30. Hence, to increase the power of our tests, we left the control variables out of the regression analyses (conform Becker, 2005). As expected, we found medium to strong correlations between different basic needs. Further, we found that gender is positively correlated with relatedness and negatively with perceived performance climate. These correlations suggest that women in our sample perceived more satisfaction of their need for personal relations at work than the men. In contrast, the men in our sample were perceiving a performance climate to a higher extent than the women.

**TABLE 2:** Means, standard deviations, and correlations with confidence intervals (*n* = 1,081).

Variable	Mean	*SD*	1	2	3	4	5	6	7	8	9	10
1. Vigor (T2)	5.22	1.15										
2. Emotional exhaustion (T2)	2.62	1.20	−0.55**									
3. Autonomy (T1)	5.06	0.81	0.41**	−0.43**								
4. Relatedness (T1)	5.49	0.80	0.40**	−0.34**	0.54**							
5. Competence (T1)	5.25	0.89	0.47**	−0.36**	0.61**	0.55**						
6. Mastery climate (T1)	3.61	0.77	0.36**	−0.31**	0.48**	0.49**	0.54**					
7. Performance climate (T1)	1.94	0.65	−0.10**	0.21**	−0.30**	−0.30**	−0.20**	−0.28**				
8. Age (T1)	45.35	10.30	0.09**	−0.06*	0.10**	−0.01	0.08*	0.05	0.03			
9. Education level (T1)	3.04	0.50	0.01	0.06	−0.05	−0.06	−0.01	−0.02	0.03	−0.04		
10. Gender (T1)	1.25	0.43	−0.03	0.05	0.05	0.12**	0.05	0.01	−0.08*	−0.17**	0.05	
11. Tenure (T1)	7.67	7.97	−0.05	0.03	−0.02	0.01	−0.03	−0.11**	0.02	0.46**	−0.06	−0.05

SD, standard deviation. *p < 0.05, **p < 0.01.

### Confirmatory Factor Analysis Results

The CFA assumed a seven-factor structure to have a better fit than all other model specifications (Table 3). Moreover, the modification indices indicated that model fit could be further improved by allowing the correlation of some of the error terms that pertain to the same latent factor. The improved fit is shown in Model 8, Table 3.

**TABLE 3:** Confirmatory factor analyses and fit indices.

Model	Factors	χ^2^	*df*	χ^2^/*df*	CFI	TLI	GFI	RMSEA	SRMR	Δχ^2^
1	Seven-factor	3.439.508	839	4.10	0.867	0.857	0.86	0.054	0.057	
2	Six-factor	5.277.324	845	6.25	0.774	0.758	0.75	0.070	0.072	1,837.8***
3	Six-factor	4.982.768	845	5,90	0.789	0.774	0.79	0.067	0.061	1,543.3***
4	Five-factor	3.971.202	850	4.67	0.841	0.831	0.83	0.058	0.061	531.69***
5	Four-factor	5.775.862	854	6.76	0.749	0.734	0.72	0.073	0.075	2,336.3***
6	Three-factor	7.273.930	857	8,49	0.672	0.655	0.68	0.083	0.077	3,834.4***
7	One-factor	10.559.945	860	12.28	0.505	0.480	0.59	0.102	0.097	7,120.4***
8	Seven-factor modified	2,676.073	831	3.22	0.906	0.898	0.89	0.045	0.052	763.44***

Model 1 is our measurement model in which items were loaded onto their respective factors (i.e., perceived mastery climate, perceived performance climate, the need for autonomy, the need for competence, the need for relatedness, emotional exhaustion, and vigor). Model 2 is a six-factor model in which perceived mastery climate and perceived performance climate items were loaded onto one factor, and all other measures were loaded onto their respective factors. Model 3 is a six-factor model in which emotional exhaustion and vigor items were loaded onto one factor, and all other measures were loaded onto their respective factors. Model 4 is a five-factor model in which the need for autonomy, the need for competence, and the need for relatedness items were loaded onto one factor, and all other measures were loaded onto their respective factors. Model 5 is a four-factor model in which perceived mastery climate and perceived performance climate items were loaded onto one factor; the need for autonomy, the need for competence, and the need for relatedness items were loaded onto one factor, and all other measures were loaded onto their respective factors. Model 6 is a three-factor model in which perceived mastery climate and perceived performance climate items were loaded onto one factor; the need for autonomy, the need for competence, and the need for relatedness items were loaded onto one factor, and emotional exhaustion and vigor items were loaded onto one factor. Model 7 is a one-factor model in which all measure items were loaded onto one factor. Model 8 is our measurement model in which items were loaded onto their respective factors (i.e., perceived mastery climate, perceived performance climate, the need for autonomy, the need for competence, the need for relatedness, emotional exhaustion and vigor), and error terms were allowed to correlate if they pertained to the same latent factor. ***p < 0.001.

### Structural Equation Modeling Results

To simultaneously examine the mediating influence of the three basic needs, we conducted a series of linear regressions. Table 4 presents the results of four mediation models. Table 4 shows a significant direct influence of perceived mastery climate on vigor (β = 0.12, *p* < 0.001). In addition, the total indirect influence of the mediation model for all basic needs together is significant (β = 0.278, *p* < 0.001). The 95% bias-corrected confidence interval (CI) for the total influence (derived from 1,000 bootstrap samples) did not contain zero, 95% CI = [0.235, 0.321]. Moreover, the analysis showed significant positive indirect influence of all three basic needs. The largest influence was from competence (β = 0.144, CI = [0.103, 0.184]). This pattern of results is consistent with a mediating influence of all three basic needs, thereby supporting Hypothesis 1, which predicted that basic needs satisfaction mediates the positive relationship between a perceived mastery climate and vigor.

**TABLE 4:** Bootstrap analyses of the magnitude and significance of direct, indirect, and total influence.

Predictor variable	Parallel mediator variables	Dependent variable	Standardized indirect influence	95% CI
	Autonomy		0.064	[0.031, 0.097]
Mastery climate	Relatedness	Vigor	0.070	[0.032, 0.109]
	Competence		0.144	[0.103, 0.184]
Total indirect influence			0.278	[0.235, 0.321]
Direct influence mastery climate → vigor			0.12	[0.023, 0.21]
	Autonomy		−0.048	[−0.071, −0.025]
Performance climate	Relatedness	Vigor	−0.053	[−0.076, −0.030]
	Competence		−0.059	[−0.081, −0.037]
Total indirect influence			−0.160	[−0.195, −0.124]
Direct influence performance climate → vigor			0.10	[0.001, 0.21]
	Autonomy		−0.134	[−0.173, −0.095]
Mastery climate	Relatedness	Emotional exhaustion	−0.050	[−0.089, −0.011]
	Competence		−0.051	[−0.090, −0.011]
Total indirect influence			−0.235	[−0.278, −0.191]
Direct influence mastery climate → emotional exhaustion			−0.12	[−0.23, −0.008]
Performance climate	Autonomy		0.083	[0.055, 0.111]
	Relatedness	Emotional exhaustion	0.032	[0.007, 0.056]
	Competence		0.024	[0.009, 0.040]
Total indirect influence			0.140	[0.108, 0.171]
Direct influence performance climate → emotional exhaustion			0.12	[0.012, 0.24]

The analysis, with respect to Hypothesis 2, shows a significant negative influence of perceived mastery climate on emotional exhaustion (β = −0.12, *p* = 0.039). The total indirect influence of the mediation model for all basic needs together is significant (β = −0.235, *p* < 0.000). The 95% bias-corrected CI for the total influence (derived from 1,000 bootstrap samples) did not contain zero, 95% CI = [−0.278, −0.191]. The analysis showed significant negative indirect influence of all three basic needs. The largest influence was from autonomy (β = −0.134, CI = [−0.173, −0.095]). This pattern of results is consistent with a mediating influence of all three basic needs, thereby supporting Hypothesis 2, which predicted that basic needs satisfaction mediates the negative relationship between a perceived mastery climate and emotional exhaustion.

With respect to Hypothesis 3, we found a significant negative total influence of performance climate on vigor (β = −0.18, *p* = 0.001). Interestingly, we see a positive direct influence of performance climate on vigor (β = 0.10, *p* = 0.045). These results form a fascinating pattern, as the negative influence of performance climate on vigor is completely due to the indirect mechanism operating through the mediator variables: the total indirect influence of the mediation model for all basic needs together is significant and negative (β = −0.160, *p* < 0.000). The 95% bias-corrected CI for the total influence (derived from 1,000 bootstrap samples) did not contain zero, 95% CI = [−0.195, −0.124]. Further, the analysis showed significant negative indirect influence of all three basic needs. The largest influence was from competence (β = −0.059, CI = [−0.081, −0.037]). The direct pathway is in the opposite direction of the total influence, but it is not strong enough to cancel out the negative influence of the indirect pathways (MacKinnon et al., 2007). This pattern of results is consistent with a mediating influence of all three basic needs, thereby supporting Hypothesis 3, which predicted that basic needs satisfaction mediates the negative relationship between a perceived performance climate and vigor.

Finally, we found a significant positive direct influence of a perceived performance climate on emotional exhaustion (β = 0.12, *p* = 0.039). In addition, the total indirect influence of the mediation model for all basic needs together is significant (β = 0.140, *p* < 0.000). The 95% bias-corrected CI for the total influence (derived from 1,000 bootstrap samples) did not contain zero, 95% CI = [0.108, 0.171]. Further, the analysis showed significant positive indirect influence of all three basic needs. The largest influence was from autonomy (β = 0.083, CI = [0.055, 0.111]). This pattern of results is consistent with a mediating influence of all three basic needs, thereby supporting Hypothesis 4, which predicted that basic needs satisfaction mediates the positive relationship between a perceived performance climate and emotional exhaustion.

In summary, we found that a perceived mastery climate is positively and significantly related to each of the three basic needs. Similarly, a perceived performance climate is negatively and significantly related to each of the three basic needs. Moreover, the results indicate that the basic needs are significantly associated with both measures of energy, negatively with emotional exhaustion and positively with vigor.

## Discussion

Drawing from AGT (Nicholls, 1989; Ames, 1992b), SDT (Deci and Ryan, 2000; Gagné and Deci, 2005), and theory on psychological energy (Schaufeli and Salanova, 2007; Shirom, 2010), we propose that the perceived motivational climate, through employee psychological need satisfaction, plays a pivotal role in predicting employee energy (i.e., vigor and emotional exhaustion) at work. We found support for these theoretical expectations. As our results illustrate, a mastery climate facilitates employee need satisfaction and subsequently increased future vigor, while reducing future emotional exhaustion. On the other hand, a performance climate contributed to thwart need satisfaction and, in turn, depleted future vigor and increased future emotional exhaustion. The satisfaction or thwarting of the need for competence had a strong influence on either enhancing or reducing vigor. When it came to enhancing or reducing emotional exhaustion, the need for autonomy had a stronger influence. Our study makes several theoretical and practical contributions.

### Theoretical and Practical Contributions

One of our most important theoretical contributions is to place a spotlight on the importance of basic psychological needs as mediators in the perceived motivational climate–energy relationship in the work setting. Although other studies (e.g., Reinboth and Duda, 2006; Van den Broeck et al., 2008; Quested and Duda, 2009, 2011) have explored the direct relationship between the perceived motivational climate and need satisfaction, as well as the direct relationship between need satisfaction and burnout, to our knowledge there is no other study that has examined how the perceived motivational climate at work predicts employee vigor/emotional exhaustion through employees’ satisfaction of basic psychological needs. Given how detrimental energy depletion is for employees and their functioning at work (Fritz et al., 2011), this is a weakness in the extant literature. Thus, understanding the process that leads to energy enhancement or energy depletion is vital.

Our results extend the current literature by showing that job demands and resources do not play the only relevant role in triggering energy enhancing or impairing processes at work (Van den Broeck et al., 2008); the perceived motivational climate also seems to activate such processes. Specifically, we found that a perceived mastery climate triggered an energy-enhancing process by increasing the vigor of employees through satisfaction of needs. By contrast, a perceived performance climate activated an energy-depletion process, by increasing emotional exhaustion through a reduction of need satisfaction. This suggests that employees who perceive that the criteria of success involve mastery, effort, development, cooperation, and learning (mastery climate) are more likely to experience a general feeling of proficiency (competence), interpersonal belongingness (relatedness), and volition (autonomy), which in turn explains why they feel less emotionally exhausted and more vigorous at work.

Our results suggest that, because of the controlling features of a performance climate (e.g., forced interpersonal rivalry among colleagues, normative ability comparison), employees may have their psychological needs thwarted, which means that they experience low levels of competence, psychological freedom, and choice, as well as low connectedness to others. In turn, employees experience more feelings of exhaustion and low energy (low vigor). These findings contribute to the occupational health psychology literature by shedding light on the energy-enhancing/depleting process underlying the relationship between the perceived motivational climate and employee energy.

Still, it should be noted that although not evident in our data, theory and other data sets in sport, education, and work (e.g., Roberts, 2012; Skerlavaj et al., 2017; Roberts and Nerstad, 2020) suggest that for some people being in a performance climate is motivating and performance enhancing. But these people are very confident of their competence and seek to demonstrate their superiority through competing with others. While the perception of high ability and success lasts, these people seek challenging tasks and revel in demonstrating their superior competence. But, the perception of high ability is fragile and may waver when the individual enters a more elite competitive environment. Then these people are likely to adopt maladaptive achievement strategies, namely, to seek easy tasks, reduce effort, or give up in the face of difficulty (e.g., Dweck and Elliot, 1983).

Further, such a contribution involves the finding that satisfaction or reduction of the need for competence had a stronger influence on employees’ energy than the other two needs by enhancing or reducing vigor. This finding suggests that the satisfaction of the need for competence may be most important for enhancing employees’ vigor, whereas if this need is thwarted, they are more likely to experience reduced vigor. This finding, to some extent, aligns with research on hip-hop dancers, suggesting that satisfaction/thwarting of the need for competence enhanced/impaired positive affect (Quested and Duda, 2009).

Our results also show that the satisfaction/thwarting of the need for autonomy more strongly influenced an enhancement/reduction of emotional exhaustion, compared to the needs for relatedness and competence. This finding is contrary to what Quested and Duda (2011) found among a sample of dancers—they did not find support for the important role of the need for autonomy with respect to emotional exhaustion. This challenges the SDT-grounded conceptualization of basic needs as essential for optimal functioning. Our results rather support the contention of SDT (Deci and Ryan, 2000) that the needs are essential for individual functioning and well-/ill-being; in our study, the needs for autonomy (for emotional exhaustion) and competence (for vigor) were found to be particularly essential. These findings add to previous research on psychological needs and psychological energy, because we clarify the influence of each discrete psychological need on energy and not only the influence of a total need satisfaction score on energy, which has been a limitation of previous research (e.g., Van den Broeck et al., 2008).

Second, our study contributes to the literature on psychological energy by drawing on two strong and unifying motivational theories—AGT and SDT (cf. Meyer and Gagné, 2008). By doing so, we have contributed to further clarify the environmental determinants and underlying psychological mechanisms of employee energy, from a motivational point of view (Meyer and Gagné, 2008; Shirom, 2010; Aronsson et al., 2017). The concepts of motivational climate and need satisfaction are particularly attractive, because they allow leaders and their organizations to learn about the environmental conditions under which the three needs may be satisfied and, in turn, promote employee energy. The relevance of the empirical links between AGT and SDT has previously been recognized and tested (Ntoumanis, 2001); however, their combined relevance as theoretical frameworks to understand the energy enhancement/depletion process has, to our knowledge, not yet been clarified sufficiently in a work setting. This also directly relates to concerns emphasized in the literature about how organizations lack understanding of human sustainability (Pfeffer, 2010; Fritz et al., 2011). This means that organizations cannot expect employees to perform at a consistently high level if employees cannot sustain their energy over long periods of time. Our study adds to the literature by clarifying the role of motivational determinants and mechanisms in sustaining or depleting employee energy over time.

Although our study was not on investigating gender differences with respect to energy-enhancing and energy-depletion processes, the uneven gender distribution in our sample requires attention as it may have affected our results. The preponderance of male participants in the study may possibly be explained by the work areas represented in the union. Still, the uneven gender distribution in our sample may be interpreted with respect to previous empirical evidence. A recent study among health professionals in Europe showed that women report lower scores on autonomy and competence compared to men (Gómez-Baya et al., 2018). With respect to work engagement (where vigor is one subdimension), the results regarding gender are inconsistent (Schaufeli et al., 2006). In Norwegian, Finnish, German, and Belgian samples, men had higher engagement compared to women, whereas in Spanish and South African samples, women reported higher work engagement scores than men. In Canadian, French, and Australian samples, no gender differences in engagement have been found. Despite these findings, some authors have suggested that work engagement may be a gendered construct where it is easier for men to be engaged at work compared to women, possibly as a result of the so-called gendered organization (Banihani et al., 2013). Further, meta-analytical evidence has shown that women are slightly more emotionally exhausted than men (Purvanova and Muros, 2010). These findings suggest that there is a need for additional research to clarify the relevance of our findings in more gender-balanced samples, including in other work areas than the ones represented in our study.

Our research has practical value for leaders, employees, and their organizations, as it provides guidance on how to enhance healthy employee functioning through the construction of working environments that promote the satisfaction of basic psychological needs and, in turn, enhance employees’ psychological energy at work over time. According to our results, to ensure that a work environment is health promoting, it seems useful to facilitate a mastery climate. This is particularly so, because such a climate facilitates supportive features for employees to experience the satisfaction of their basic psychological needs for connectedness to others, volition, and competence (Reinboth and Duda, 2006). Although there is little evidence concerning the best way to foster a mastery climate in an organizational setting (DeShon and Gillespie, 2005), findings from sports and education domains may provide some clues as to what may be beneficial: (a) designing meaningful and interesting tasks that include variety, challenge, and control; (b) giving each individual opportunities to make choices and to participate in decision making, as well as the self-determination to decide upon strategies for completing the task; (c) giving thorough consideration to how outcomes and striving behaviors are recognized by avoiding rewards and recognition that are perceived as bribes or methods of control (Deci and Ryan, 2000); (d) encouraging appreciation of the differences between individuals by treating everyone in a similar way; (e) evaluating each individual privately based on his/her progress, mastery, creativity, and effort; and (f) managing time by allowing those individuals who need more time to develop the necessary skills to perform at a higher level to have more time (Ames, 1992b; Roberts et al., 2018). Moreover, the basic psychological needs concept has practical value, in that it represents a vital mechanism that may be fruitful in understanding how to facilitate a more long-term sustainability of employee energy at work (cf. Van den Broeck et al., 2008, 2016; Pfeffer, 2010; Vansteenkiste et al., 2020).

## Limitations and Directions for Future Research

Although our results are promising, several limitations must be addressed. As the overall study was reliant on self-report measures, the results may have been influenced by common method variance. However, our variables of interest, vigor, and emotional exhaustion, are difficult for others to assess, and in such cases, the use of self-reports is considered to be warranted (Conway and Lance, 2010; Kim et al., 2013). It has been shown that individuals can detect differences in their own mood and behavior better than external raters (Lance et al., 1992), who usually fall back on general impressions (Lance et al., 1994). Nevertheless, we decided to limit possible effects of common method bias by gathering data about our dependent variable at a later time point (T2) than we gathered data on the predicator and mediating variables. In addition, we followed Podsakoff et al. (2003) recommendations regarding how to control for method biases through study design, such as by requesting honest answers and ensuring anonymity. Moreover, all correlations were less than the threshold of 0.70 (Tabachnick and Fidell, 2001), indicating that the likelihood of multicollinearity is low. In addition, we conducted a CFA containing all items in our model, which should produce a good fit of a single factor if common method bias is likely in our data (Podsakoff and Organ, 1986). The GFIs (χ^2^ = 10559.945, *df* = 860, χ^2^/*df* = 12.28, CFI = 0.505, TLI = 0.480, GFI = 0.59, RMSEA = 0.102, SRMR = 0.097) indicate a poor fit for the single-factor model and an acceptable fit for the model that contains all construct variables (χ^2^ = 2676.073, *df* = 831, χ^2^/*df* = 3.22, CFI = 0.906, TLI = 0.898, GFI = 0.890, RMSEA = 0.045, SRMR = 0.052), which suggests that bias from common method variance is unlikely and that our measures provided sufficient discriminant validity (Podsakoff et al., 2003, 2012). Nevertheless, future studies may want to think of alternative research designs to further minimize the risk of bias.

All fit indexes for the seven-factor model were acceptable, except for the TLI and GFI indexes, which are recommended to be 0.90 or greater (Marsh, 2007). It should, however, be noted that because of the sensitivity of the GFI index, it has become less popular, and it has been recommended that it should not be applied (Sharma et al., 2005). Further, conventional CFA goodness of fit criteria (e.g., CFI, TLI, RMSEA) have been argued to be too restrictive when applied to most multifactor (e.g., 5–10 factors) rating instruments (Marsh et al., 2004; Marsh, 2007). Consequently “it is almost impossible to get an acceptable fit” and “conventional” rules of thumb about acceptable fit are “too restrictive” (Marsh et al., 2004, 325). Still, the TLI and GFI fit of our model may represent a potential limitation.

Another limitation is that our sample included only engineers and technologists, although representing various work areas. Most occupations remain gender-typed, where men frequently are employed in professions that fit stereotypes about male gender roles (e.g., well-paid jobs, physically demanding jobs), whereas women often are employed in professions that fit stereotypes about female gender roles (e.g., nurturing/caregiving jobs) (Purvanova and Muros, 2010). For example, females are underrepresented in occupations such as engineers and architects (Purvanova and Muros, 2010). Differences according to the scope of profession may have an impact particularly when considering that the domination by one gender in a particular profession is likely to create negative experiences for members of the underrepresented gender. Hunt and Emslie (1998) found that women reported poorer self-assessed health and higher psychological distress in male-dominated occupations compared to female-dominated occupations. This knowledge may limit the extent of generalizability of our results to other occupations. To strengthen the external validity (Cook and Campbell, 1979) of our findings, a more extensive study is necessary, one that includes organizations with other occupational groups and that accounts for men and women in atypical versus typical occupations (Purvanova and Muros, 2010).

Further, the total sample consisted of 75% men. Although this uneven gender distribution was in line with the overall union statistics (78% male members), our results should be interpreted with this unbalanced composition by gender in mind. This gender unbalance in our sample as well as in the total union may possibly be explained by the various represented work areas. Given that the literature in general shows gender differences in relation to perceived motivational climate, basic psychological needs, and in terms of vigor and emotional exhaustion (e.g., Kavussanu and Roberts, 1996; Schaufeli et al., 2006; Purvanova and Muros, 2010; Gómez-Baya et al., 2018), it is important that future research explores the energy-enhancing and energy-depletion processes, which are investigated in our study. This is needed in more gender-balanced samples as well as across other work areas than the ones included in our study. It is particularly relevant as it is not possible to ignore that it is probable that women experience more emotional exhaustion and health problems due to the multiplicity of roles that they often must fulfill^2^ (Purvanova and Muros, 2010; Gómez-Baya et al., 2018).

Although the focus of this research was to study the psychological motivational climate, it should be noted that this study was conducted through a union; therefore, participants were spread across various organizations from different parts of Norway, making it impossible to examine whether climate perceptions were shared among the employees in a given workplace. Although we believe that the motivational climate within an organization constitutes an important aspect of its psychological climate (Parker et al., 2003), we suggest that future organizational research could benefit from exploring the group level of the motivational climate.

It also seems important to emphasize that some of the standardized β coefficients in the current study were significant, but rather low. A large sample size may typically result in significant coefficients representing the association between relevant variables, but it is up to the researcher to consider their meaningfulness. We believe that our results are meaningful and that the low β coefficients may be due to the time interval of 7 months. The interpretation of the results presented in our study should be viewed in light of this limitation.

The antecedents of the motivational climate at work represent another vital aspect that may be important for future research. The leader of a given workplace has often been put forward as the main architect of the motivational climate (Ames and Ames, 1984; Ames, 1992a; Dragoni, 2005). Significant others (e.g., colleagues) and/or organizational practices may also play an important role in influencing such a climate (Janssen and Van Yperen, 2004; Roberts et al., 2018). As we know considerably less about the antecedents of the climate than we do about their outcomes (Kuenzi and Schminke, 2009), we suggest that one fruitful line of inquiry for future research may be to investigate human resource management practices and/or job designs as prospective determinants of the perceived motivational climate at work. Future research may thereby contribute suggestions for some specific criteria for facilitating a mastery climate at work and thus further explore where the energy-enhancing/depleting process starts.

## Conclusion

Employees differ in the amount of energy they display at work (i.e., vigor and exhaustion). Energy depletion at work has been described as a human energy crisis (Fritz et al., 2011). Drawing on motivational theory (AGT and SDT), we proposed that the perceived motivational climate at work is likely to influence the level of employee energy and that basic psychological needs mediate the relationship. We found that a mastery climate activates an energy enhancing process through the satisfaction of basic psychological needs—thus preventing an energy crisis. A performance climate, on the other hand, was found to generate an energy-depletion process through thwarting of basic psychological needs, thus triggering an energy crisis. Our study thereby adds to the occupational health psychology and psychological energy literature.

## Data Availability Statement

The datasets generated for this study are not publicly available due to the regulations set by the Norwegian Center for Research Data. This is to protect respondent confidentiality and participant privacy. Requests to access the datasets should be directed to CN, chrner@oslomet.no.

## Ethics Statement

Ethical review and approval was not required for the study on human participants in accordance with the local legislation and institutional requirements. The patients/participants provided their written informed consent to participate in this study.

## Author Contributions

CN, AR, and GR contributed to the conception and design of the study. CN organized the database. MC performed the statistical analysis. CN wrote the first draft of the manuscript. MC and GR wrote sections of the manuscript. All authors contributed to manuscript revision, read and approved the submitted version.

## Conflict of Interest

The authors declare that the research was conducted in the absence of any commercial or financial relationships that could be construed as a potential conflict of interest.

## Footnotes

1 The union’s registration system for members’ e-mail addresses is never completely up-to-date. Although the questionnaire was distributed to 33,275 members, the union received delivery failure e-mail messages from several members; however, it did not keep a record of the number of delivery failures. We therefore cannot be certain about the match between the number of surveys and the respondents who received them.

2 We would like to thank one of the reviewers for pointing to this.
